# Modelling developments in consciousness within a multidimensional framework

**DOI:** 10.1093/nc/niae026

**Published:** 2024-06-17

**Authors:** Mads Jørgensen Hansen

**Affiliations:** Department of Philosophy and History of Ideas, School of Culture and Society, Aarhus University, Aarhus 8000, Denmark

**Keywords:** consciousness, multidimensional models, temporal profiles, disorders of consciousness

## Abstract

A recent advancement in consciousness science has been the introduction of a multidimensional framework of consciousness. This framework has been applied to global states of consciousness, including psychedelic states and disorders of consciousness, and the consciousness of non-human animals. The multidimensional framework enables a finer parsing of both various states of consciousness and forms of animal consciousness, paving the way for new scientific investigations into consciousness. In this paper, the multidimensional model is expanded by constructing temporal profiles. This expansion allows for the modelling of changes in consciousness across the life cycles of organisms and the progression over time of disorders of consciousness. The result of this expansion is 2-fold: (i) it enables new modes of comparison, both across stages of development and across species; (ii) it proposes that more attention be given to the various types of fluctuations that occur in patients who are suffering from disorders of consciousness.

## Introduction

Consciousness science has recently seen the introduction and development of several multidimensional frameworks of consciousness (Bayne and Hohwy 2016, Bayne et al. 2016, 2017, Jonkisz et al. 2017, Sergent et al. 2017, Bayne and Carter 2018, Fazekas and Overgaard 2018, Birch et al. 2020, McKilliam 2020, Dung and Newen 2023, Huang et al. 2023). In this paper, I focus specifically on multidimensional models of global states of consciousness (GSC) (Bayne et al. 2016, 2017, McKilliam 2020) and non-human consciousness (Birch et al. 2020).

Multidimensional frameworks allow us to move away from a unidimensional conception of consciousness, a single dimension along which we may order conscious entities from least to most conscious (Birch et al. 2020, p. 709). By understanding consciousness as varying along several dimensions, we obtain a fine-grained understanding of the many ways in which consciousness may take shape.

This paper accepts the multidimensional approach to consciousness, but expands this framework by constructing temporal profiles. Consciousness is not only multidimensional, but changes over time, and expanding the framework enables us to model the life history of organisms in terms of their changes in consciousness. By paying greater attention to changes in consciousness, new modes of comparing the consciousness of one organism to that of another become possible. We may ask how great the variation in consciousness is over time, what the velocity of that variation is, and inquire into the dependency relations among the many dimensions.

I will argue that some patterns of change in disorders of consciousness (DoCs), invisible in non-temporal representations of consciousness, may function as kind-identifying patterns of DoCs. With this approach I will propose shifting the focus on DoC research from mainly looking for stable indicators of consciousness in a patient whose conscious states fluctuate, to taking those fluctuations as information about kinds of DoC. The expanded framework indicates that we may define at least some DoCs by their temporal extension. Similar to how a symphony is not wholly present at one moment in time, certain DoCs may also not be entirely present at a given moment. Increasing attention to changes in consciousness creates the possibility of finding new patterns that are diagnostically and prognostically useful.

The paper is structured as follows. In Section ‘Multidimensional frameworks of consciousness’, I introduce the multidimensional framework for GSC, McKilliam’s (2020) proposed capacities account of GSC, and the multidimensional framework of animal consciousness. In Section ‘Changes in profiles’, I criticize the capacities account, and argue that it neglects the development of consciousness-related capacities. In Section ‘Case 1: butterfly life cycles’, I expand the multidimensional framework by constructing temporal profiles. I use the butterfly’s life cycle to illustrate the philosophical and scientific advantages of modelling the development of consciousness. In Section ‘Case 2: disorders of consciousness’, I turn from animal consciousness to DoC. I present an overview of DoC and some of the ways that the conditions of patients with DoCs may fluctuate. I argue that modelling developments of consciousness creates the possibility of defining DoCs by their patterns of fluctuation.

## Multidimensional frameworks of consciousness

### Global states of consciousness

GSC is one’s overall state of consciousness. Examples include waking awareness, rapid eye movement (REM) dreaming, meditation, being in the minimally conscious state and being high on psilocybin. ‘[C]onscious states can be thought of as global dimensions of consciousness that modulate both the kinds of contents that can enter consciousness and the way in which those contents can be used by the organism for cognitive and behavioural control’ (Bayne and Carter 2018, pp. 1–2).

McKilliam (2020) argues that GSC are not phenomenal states in the sense of ‘what it’s like’ to be conscious (Nagel 1974). Instead, McKilliam (2020) argues that GSC are constituted by a set of what he calls consciousness-related capacities, i.e. capacities that cannot be specified without reference to phenomenal consciousness, e.g. the capacity for visual colour experiences. This is consistent with the ideas of Fortier-Davy and Millière (2020) who see the dimensions of GSC as ‘dispositional properties of conscious subjects’ (2), and Soteriou (2019), who defines conscious wakefulness in terms of the uninhibited and synthesized exercise of various capacities that make a temporal point of view possible (132).

McKilliam (2020) argues that a specific GSC is captured by the set of consciousness-related capacities of a system that are currently online. To distinguish between a system and the states of a system, McKilliam gives the following example. Washing machines are systems, but the washing machine may operate in many different states, where states are the various programs the machine can run. When the washing machine is running one program, the other possible programs it can run are offline. Here, an offline program is a possible state in which the system may be, but currently is not. McKilliam provides the following definition of GSC, which I adopt in this paper:

**Global states of consciousness**: states of creatures (systems) that regulate (i) the range and quality of conscious contents the creature is capable of experiencing while in that state, (ii) the range of cognitive systems into which those contents can be mobilized while in that state, and (iii) the range of attentional capacities the creature has while in that state. (McKilliam 2020, p. 14).

In the capacities account, a GSC is defined in relation to the capacities that are not online, i.e. a GSC is defined in relation to the types of experiences a subject can have but is not having. False awakenings and true awakenings are different GSC (dreaming and wakefulness, respectively), even if they are equivalent phenomenal states, because they differ in the types of experiences the subject can have in either state.

#### Which dimensions go into the multidimensional framework?

**Figure 1:**
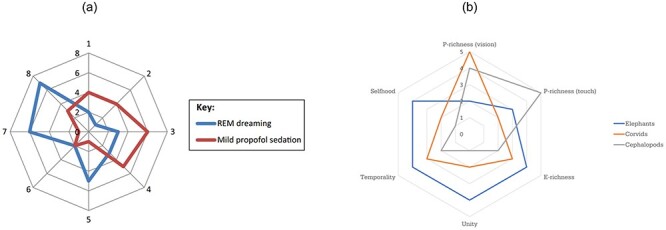
(a) A multidimensional model of various GSC (from Bayne et al. 2016) (b) A multidimensional model of animal consciousness, with the various sentience profiles of various organisms (from Birch et al. 2020)

The multidimensional framework of GSC is in its infancy, and therefore, the jury is still out on the dimensions along which GSC may vary. Bayne and Hohwy (2016) and Bayne et al. (2016) argue that even though GSC and conscious content are distinct, conscious content does play some role in what constitutes a GSC. GSC vary in how much content they allow to enter consciousness; for example, during wakefulness, you are aware of the details of an object to a high degree, as opposed to when you are in a drowsy state, where the content is less fine-grained (Fig. 1a).

Fazekas and Overgaard (2016, 2018) argue that conscious perception should be split into three dimensions: levels of processing content, intensity of content, and precision of content. Bayne and Hohwy (2016) argue for a dimension they call ‘attentional structure’, which captures the ways in which ‘the field of consciousness is distributed between what falls within focal attention and what falls outside it’ (67). Bayne et al. (2016) propose functional dimensions that are separate from the dimensions that relate to content. Here, a GSC may vary along several dimensions tied to cognitive and behavioural capacities. For example, fully functioning humans in wakeful states will score high on the functional dimensions, whereas people who suffer from unresponsive wakefulness syndrome (UWS), also called the vegetative state (VS), will have low scores, because of their inability to express certain behaviours. Others, such as Jonkisz et al. (2017), distinguish among four dimensions—phenomenological, semantic, physiological, and functional—and Pautz (2019) distinguishes among five dimensions (which he calls ‘levels’): intensity, complexity, determinacy, access, and richness of experiential repertoire. Both Bayne et al. (2017) and Sergent et al. (2017), when speaking specifically about DoC, distinguish among eight dimensions, and Dung and Newen (2023), who focus specifically on consciousness across species, distinguish among 10 dimensions.

In the literature there is some overlap among the various dimensions, and some dimensions are more fine-grained versions of others. It should be noted that any dimension specifically related to phenomenality, such as the one in Jonkisz et al.’s (2017) account, is excluded from the capacities account, as GSC are not phenomenal states. The specific dimensions along which GSC may vary and the extent to which such dimensions should be more finely parsed is a matter for future research, but practicality does play a role, as Birch et al. note (Birch et al. 2020): ‘If our goal were to capture all interesting variation in conscious states, we would never have enough dimensions. We have to be pragmatic’ (797).

To recap, GSC are not phenomenal states but the set of consciousness-related capacities that are online. Which capacities (dimensions) will be included in the model is still being worked out in the literature. I will now turn to a recent application of the multidimensional framework to non-human consciousness. Once I have done so, I will present a criticism of the capacities account to argue that it should include the development of those capacities.

### Sentience profiles

Birch et al. (2020) have recently applied the multidimensional approach to non-human organisms. They present a multidimensional model, where each dimension addresses the ways in which consciousness may vary for conscious entities (Fig. 1b). The model includes five dimensions, which Birch et al. regard, in a first approximation, as comprising the multidimensional framework of animal consciousness:

- **‘**P-richness’ or perceptual richness refers to the level of detail with which an animal consciously perceives its environment. The fine-grained quality of conscious perception may vary along several senses, such as vision, touch, olfaction, and hearing. In the model (Fig. 1b), Birch et al. have included only vision and touch, though the model may easily be expanded to include other forms of perception.
- **‘**E-richness’ or evaluative richness refers to an organism’s affective response to an experience. An affective response has either a positive or a negative valence.
- **‘**Unity’ is the degree of integration of conscious experience at a time. For humans, conscious experience is highly unified, in that there is a single ‘you’, not several ‘yous’ with conscious experiences. Split-brain patients may differ from non-split-brain individuals along this dimension.
- **‘**Temporality’ refers the degree to which an animal’s experiences are integrated over time. This dimension captures the ability to experience the world as a coherent stream over time, and the ability to do ‘mental time-travel’ to recall past experiences and predict future events.
- **‘**Selfhood’ or self-consciousness refers to the conscious awareness of having a self, i.e. having a distinction between oneself and the actions produced by that self, on the one hand, and the outside world and the inputs it provides, on the other.

GSC profiles and sentience profiles overlap in their aims as both attempt to capture consciousness through consciousness-related capacities. Yet, they do differ in scope. Originally, the GSC framework (Bayne et al. 2016) was intended to illustrate that we should abandon the idea that there are levels of consciousness for human GSC, whereas the sentience framework is intended to illustrate that we should abandon the idea of levels of consciousness among conscious animals (Birch et al. 2020). The expansion to the multidimensional model that I propose in this paper is for any multidimensional model of consciousness, regardless of scope.

The multidimensional approach to consciousness, whether GSC or sentience, is an advancement in consciousness science precisely because it gives a more detailed representation of ways of being conscious. However, I argue that in its current form, the framework overlooks the pragmatic utility of modelling developments in consciousness.

## Changes in profiles

Both McKilliam (2020) and Birch et al. (2020) define the dimensions of GSC and sentience as capacities. How should we understand change in the capacities account? Capacities are fundamentally the product of an organism’s morphology and physiology. For example, if an organism has camera eyes, then that organism will score differently on the P-richness (vision) dimension than organisms with simple eyes. Some organisms radically change their morphology throughout their life cycles. This means that if we take ontogeny into account and track the morphological and physiological changes throughout an organism’s life cycle, we should expect to see changes in the organisms’ sentience profiles.

Evidently, change is an integral part of the capacities account, since GSC change according to which capacities are offline and online. But this is only one of many types of change. I will argue that there is a friction in McKilliam’s (2020) capacities account, a friction between an implicit assumption of static systems with modulating static capacities and the development and loss of those capacities.

McKilliam explains what is needed for an organism to acquire or fail to acquire a capacity, and presents three options:

- The capacity has yet to be developed/learned: some systems have the capacity to learn a language, but have yet to do so.
- The capacity cannot be acquired: some organisms, such as dogs, cannot learn to speak French.
- The capacity is off-line: some organisms have the capacity to speak, but that capacity is currently offline.

Regarding the three options, McKilliam states, ‘It is the third sense that I am interested in here. In order to understand the capacities account of global states we need to appreciate the distinction between the total set of capacities that a creature (a system) has, and the set of capacities that creature has while in a particular global state’ (McKilliam 2020, pp. 12–13).

Of the three options, it is clear that (ii) stands out because it is purely restrictive, leaving no room for change. In (i) and (iii) there is the potential for capacities while currently absent or offline, to be developed or come online, respectively. Option (i) offers the possibility of the development of, or the potential to learn, capacities that may then shift between being offline and online. Thus, for (i), change is understood as a change in the set of capacities that can go offline and online, whereas in (iii), what changes is which capacities are online and offline.

For (i) we can distinguish between developing a capacity from non-existence to existence, and the development of an existing capacity. For example, the development of eyes changes an organism’s score on the P-richness (vision) dimension from zero to non-zero. This is different from the development of colour vision from infancy to early childhood.

The friction in McKilliam’s account becomes apparent once we start to take development into account. The friction is between the idea of a stable system with capacities that are turned on and off and the developing (and later degrading) system in which the capacities themselves emerge (and later fade). This is borne out by the washing machine metaphor with its many programs that are either online or offline. Machines, unlike organisms, are non-developmental making the metaphor unsuitable for organismal consciousness. There are periods of what seems like stability in organisms, a period we might refer to as adulthood, but this stability is simply due to the level of analysis applied. There are of course practical reasons for constructing a profile of the adult stage on an organism, as that stage is where development seemingly ends. Such relative stability makes it easier to capture their sentience profile. Yet, we should note that privileging the adult stage is based on practicality, and we should be wary of drawing conclusions about other stages of a life cycle from the adult stage (Nicholson and Dupré 2018, Dupré 2021). Some species of butterfly live most of their lives as caterpillars, and therefore, privileging the adult stage would be privileging a stage of a life cycle that takes up the shorter amount of time lived.

Even though development ceases once the adult stage is reached, another process still continues: deterioration. If an organism is lucky enough to escape death by predation, it will gradually lose key features for survival that are present in early adulthood. Vision fades, certain mechanisms become less reliable, and some are lost altogether. The overall accumulation of mutations and damage alter the physiology, morphology, and behaviour of the individual, which will influence the ways in which it is sentient.

All the foregoing means that the sentience profile of an octopus in its prime is no more a representation of octopus sentience than the adult stage of a human is a representation of human *simpliciter*. It is but one frame, not the full movie, one point in time at one stage of a life cycle. Because of this I argue that it is a mistake to not incorporate the development of capacities and the changes in the extent to which these capacities can be expressed in an analysis of GSC and sentience, since by not doing so we overlook new modes of comparison within and across species. For this reason, I propose enriching McKilliam’s capacities account by making it sensitive to tracking developmental changes in the set of capacities that may go online and offline, i.e. making it possible to track how capacities develop, and track changes in the ways in which capacities that are online can be expressed.

Lastly, it is important to distinguish between tracking changes in consciousness ‘over time’ in my proposed expansion of the multidimensional framework, and the dimension of Temporality in Birch et al.’s (2020) framework. As noted, the dimension of Temporality refers to the integration of experiences over time. I argue that our model should include the development of, and the changes in, the way the dimension of Temporality (and all the other consciousness-related capacities) changes over time. For example, a human child may score low on the Temporality dimension, poorly integrating experiences over the past few months, whereas later in life it may have high Temporality, being able to integrate experiences over years and decades. In short, the capacity to integrate experiences over time may itself change over time.

In what follows, I will not discuss how to settle the question of which dimensions to include in the model, nor how to measure the extent to which an organism satisfies each dimension. Neither will I discuss how to compare one organism to another along the many dimensions. All these are important questions that future research will have to address. The focus of the rest of this paper is to promote the idea that tracking developments in consciousness has practical utility. I will demonstrate this through two case studies: the life cycle of a butterfly, and DoC. By focusing on ontogeny, I will show how modelling changes in consciousness facilitates new modes of comparison of types of consciousness. By focusing on DoC, I will show how DoC research should pay greater attention to the ways in which patients fluctuate while in those DoCs, as new DoC-defining regularities may become apparent.

## Case 1: butterfly life cycles

To illustrate the importance of ontogeny in sentience research, I use the butterfly life cycle as a case study. I am ‘not’ necessarily claiming that butterflies are sentient, though it has been argued that insects are sentient, based on the functional similarity of certain brain structures to humans (Barron and Klein 2016, Klein and Barron 2016), and a recent review of pain in insects found strong evidence suggesting insects can feel pain, during at least some stages of their life cycle (Gibbons et al. 2022). The butterfly’s dramatic life cycle, from larva to pupa and from pupa to adult, makes explicit how changes in ontogeny could result in changes in sentience profiles.

**Figure 2:**
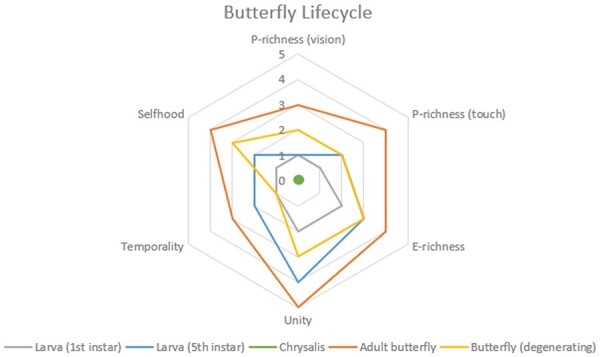
A set of sentience profiles for the various developmental stages of the butterfly life cycle. The chrysalis stage is indicated by a dot, based on the assumption that the butterfly satisfies none of the dimensions in the chrysalis stage. The extent to which each profile satisfies each dimension is not evidence-based. The profiles are constructed for illustrative purposes only, in a way that makes them easily distinguishable. It is possible that butterflies do not satisfy some dimensions or that they satisfy none

I construct a series of non-evidence based sentience profiles of a butterfly’s life history (Fig. 2). I have chosen the butterfly’s life cycle because it involves a complete metamorphosis, a not uncommon feature in the biological world as ‘More than 80% of insect species, possibly representing around 60% of all animals, undergo a particularly marked form of metamorphosis in which an ecologically inactive life stage called the pupa is interposed between the larva and the adult, during which the insect’s body is almost entirely rebuilt’ (Rolff et al. 2019, p. 1). Such a radical transformation drives home the idea that an individual’s consciousness changes over time.

Butterflies are holometabolous insects, meaning they undergo complete metamorphosis during their life cycle, the result of which is a radical difference in physiology, morphology, and behaviour between the larval and adult stages. The butterfly’s life cycle is divided into the larval (caterpillar) stage, the chrysalis (pupa) stage, and the imago (adult) stage. The larval stage consists of several stages called instars. Each instar is separated by a moult, a moult being the casting of the cuticle due to a gradual increase in body size. The order *Lepidoptera*, of which butterflies and moths are members, has between five and six instars (Chapman et al. 2013, p. 411). During the final instar, the caterpillar develops into a pupa, during which the nervous system is extensively restructured. New features develop, and others are lost, remain, or are altered.

Contrary to popular belief, *Lepidoptera* do not turn into mostly goo during pupation (Rolff et al. 2019, p. 2). For example, the brain of the sphinx moth, *Manduca sexta*, is only restructured: ‘One organ system that does not undergo histolysis during intra-puparial development is the brain, although there is considerable remodelling’ (Hall and Martín-Vega 2019, p. 7). Furthermore, even with extensive remodelling, it is possible for adult moths to retain memories acquired during the larval stage (Blackiston et al. 2008, 2015).

Metamorphosis results in changes in vision and an enlarged brain (Chapman et al. 2013, p. 428). Caterpillars have simple eyes that can detect little more than light and dark. Adults, on the other hand, have compound eyes that enable the butterfly to discern colours, shapes, and movement with greater precision than the caterpillar’s simple eyes.

The adults have more intricate neural networks, which are needed to navigate during flight. Control of flight requires a multimodal integration of sensory information, where vision needs to be able to ‘keep up’ with the speed of flight, to ensure safe landings and to avoid collisions.

Gibbons et al. (2022) found strong evidence of nociception in *Lepidoptera* at the adult stage and the first instar, little evidence of sensory integration at the first instar, and strong evidence of sensory integration in the adult stage (pp. 203–204, tables 11 and 12).

**Figure 3:**
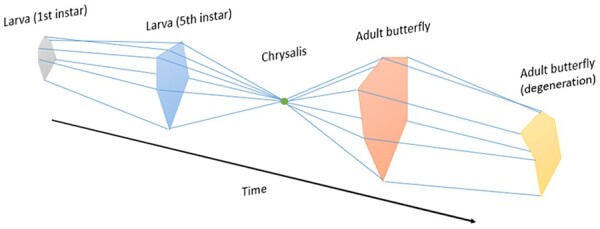
A temporal sentience profile of the butterfly life cycle, based on the assumption that during the chrysalis stage the butterfly is not sentient. The shape of the model is not evidence-based. The profiles are constructed for illustrative purposes, in a way that makes them easily distinguishable

The list of physiological and morphological differences shows how different the larval and adult butterfly modalities are. If butterflies are sentient, then these differences are accompanied by a difference in sentience. If we take the profiles of each stage of development and all the stages that lie between, and treat them as time-slices of the entire butterfly life cycle, arranging them in diachronic order, we obtain a temporal sentience profile, as pictured below (Fig. 3).

The model above is an illustration of a general idea. What the model allows us to do is to visualize experiential changes as a product of developmental changes, thereby capturing the dynamics of consciousness. The temporal sentience profile would be the product of several experiments with several butterflies of the same species at various stages of their life cycle. Such a temporal sentience profile would function as an ‘idealized’ temporal profile for all butterflies of that species. It is of course also possible to construct a temporal sentience profile for an individual butterfly. In such cases, we could construct temporal profiles that show the collapse of one or more dimensions as the result of predation or other hazards. For example, if a butterfly sustained severe damage to its eyes, this would reduce the extent to which it satisfies the P-richness (vision) dimension. This is illustrated by the last profile, the adult ‘degeneration’ stage.

Though there may be negligible differences between the consciousness-related capacities of the butterfly sexes, we should bear in mind that some species display pronounced sexual dimorphism, as in the case of the anglerfish. If this dimorphism is relevant to consciousness, then we should construct temporal sentience profiles of not only the life cycle of each species, but of each sex.

Before moving on to the pragmatic utility of the temporal sentience profile approach, let me first introduce a possible problem for the model, a solution, and a limitation.

### Splitting, merging, and temporality

Temporal models such as that described above potentially face problems when applied to entities that have either low Unity or low Temporality. If an organism has low Unity, it may house more than one consciousness. If an organism has low Temporality, it’s experiences are minimally integrated over time, which raises the question of whether we are dealing with a single conscious subject over time (I thank one of the reviewers for bringing this to my attention.). How are we to model such cases in the suggested framework? I will first discuss cases of low Unity and then turn to cases of low Temporality.

An example of low Unity is the split-brain patient. The question of whether split-brain patients house two streams of consciousness in the same body is unsettled (Pinto et al. 2017, De Haan et al. 2020, Schechter and Bayne 2021). I will set aside this debate, as it is beyond the scope of this paper, and instead discuss the consequences for the multidimensional framework if there are two streams of consciousness.

If split-brain patients house only ‘one’ conscious subject, then we would construct only one temporal sentience profile for that subject. The dimension of Unity would be satisfied to a lesser degree post-surgery than pre-surgery, as the corpus callosum, the main communication path between the two hemispheres, would have been severed. Such scenarios do not pose a problem for the temporal profile framework.

**Figure 4:**
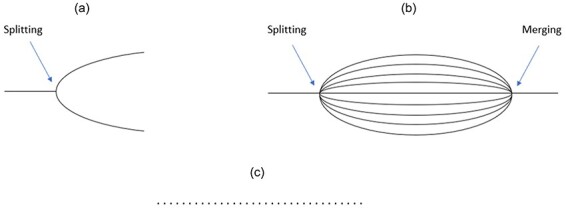
(a) Each line represents a temporal sentience profile. If split-brain patients house two subjective experiences, one tied to each hemisphere, then we may construct a temporal profile for each hemisphere. (b) If each of an octopus’s arms is conscious, then we may construct a temporal profile for each. An octopus may shift between having many subjective experiences, and only one. (c) A series of short-lived profiles ordered to indicate that they apply to the same organism

If split-brain patients house ‘two’ conscious subjects, i.e. there are two consciousnesses in the same body, we need to construct two temporal sentience profiles, one for each hemisphere (Fig. 4a). The temporal profiles might differ for each hemisphere but note that the degree of Unity for each hemisphere need not be low, though perhaps it is lower than what it was pre-surgery.

The question of whether non-human animals such as birds and octopuses can house more than one consciousness has also been raised (Godfrey‐Smith 2021). Carls-Diamante (2022) has attempted to advance the view that each of an octopus’s arms has its own consciousness. This is done on both behavioural and anatomical grounds. Amputated octopus arms exhibit grasping reflexes, flinch when pricked with a needle, and produce actions nearly identical to those seen when they are attached to the whole octopus, suggesting that the arms’ behaviour is localized in the arms, not the whole octopus. Furthermore, the arms may be capable of somatotopic mapping, and even have the capacity for memory (Carls-Diamante 2022, p. 4). If this is so, then perhaps the arms should have their own sentience profiles, separate from that of the octopus. However, Mather (2021) argues that it is still unclear how integrated octopus experience is, the evidence seeming to suggest that there is intersensory integration in the octopus’s brain, which suggests that the arms are not conscious.

We may address the case of the octopus as we did that of the split-brain patient. If there is only ‘one’ conscious subject in the octopus, then we simply construct a temporal sentience profile for the octopus life cycle. If, on the other hand, the octopus’s arms are conscious, so the octopus houses nine conscious subjects, we may construct temporal sentience profile for each arm. Presumably, the arms would have very low scores along the many dimensions in the framework.

There is a further, rather dramatic possibility, namely, that octopuses ‘merge’ their many conscious subjects. I am not here claiming that this is the case, but present the possibility, only to show that the temporal sentience profile framework can also address cases of merging, should they turn out to be possible.

One might imagine an octopus resting on the seabed, its arms exhibiting forms of autonomous behaviour, each with its own consciousness. Yet, once a predator is sighted, the octopus ‘recruits’ its arms, merging their consciousnesses to produce joint action to escape (recruitment could occur by degrees, and merge only a subset of the arms for various tasks.). When it reaches safety, and once again rests on the seabed, the octopus’s many consciousnesses could re-emerge, and fan out like the petals of a blooming flower (Fig. 4b).

We would be able to see the effect of splitting and merging in the octopus’s profile. Birch et al. (2020) gave the octopus a high score along the P-richness (touch) dimension (Fig. 1b), yet, if there is splitting, it is possible that the octopus, though perhaps not its arms, has low P-richness (touch) during periods of splitting, but attains high P-richness (touch) during merging.

I do not take a stance on whether an organism can house several conscious subjects. I only attempt to show that the proposed framework can model such scenarios. If there are several conscious subjects in the same body, we may construct temporal sentience profiles for each.

It is crucial to note that this discussion of the possible splitting and merging of the octopus’s consciousness is tied to a specific temporal scale. In Fig. 3, I present a temporal sentience profile at the ontogenetic scale, which shows how capacities develop and change the extent to which they are satisfied throughout the butterfly’s life cycle. But we can produce such temporal profiles for smaller timescales, from one that covers a sleep cycle with its many stages, to the timescale of a single behavioural change, as illustrated in the case of the octopus (Fig. 4b).

We can now talk about entities with low Temporality. Again, if an entity has very low Temporality, then perhaps there is a replacement of subjects over time. If a subject exists for only an instant, then we cannot construct a temporal sentience profile for that subject. A single profile that captures the moment the subject exists would be enough. Temporal sentience profiles are models that capture the timeline of a conscious subject. The subject may be conscious for only a single moment, which makes a single profile appropriate, or it may be conscious for several decades, which would call for a temporal profile. In the latter case we may still order the different profiles if they occur in the same organism though with no connecting lines. This would give us a model of an organism with a potentially high turnover of conscious subjects (Fig. 4c).

### New modes of comparison

By modelling the changes in consciousness-related capacities over time, we can begin to see how such capacities develop and in what way the extent to which they are satisfied changes. Perhaps some capacities are present at early stages and absent in others. Furthermore, if we hold that a specific kind of sentience profile has consequences for what the optimal approach to an animal’s welfare is, then we need to track the changes in its profile, in order to adapt our welfare measures. For example, Dung (2023) claims that some dimensions of the sentience model are of greater importance to animal welfare than others, such as E-richness and Temporality. If this is the case, it becomes paramount for research into animal welfare to discover when such dimensions come online or are expressed to their highest degree.

Yet, this temporal model not only provides a more detailed representation of consciousness than the previous version. By constructing temporal sentience profiles, ‘new’ ways of comparing sentient entities become available both within and across species. What becomes possible is the potential to compare an organism’s degree of variation and velocity of change in consciousness over time, as well as the potential to uncover the dependency relations between the dimensions.

#### Degree of variation

Suppose we constructed temporal sentience profiles for butterflies and for Greenland sharks. As we know, we can compare the two organisms along single dimensions at one point in time. Yet, we can also compare the two organisms according to how much they vary over time across those dimensions. Some organisms’ sentience profiles may be rather stable throughout their life cycle, whereas others’ may expand and contract throughout development. This change in the extent to which each dimension is satisfied may take place within a limited area, or there may be a marked difference between several developmental stages. It may become apparent that the butterfly’s consciousness life cycle is more ‘tumultuous’ than that of the Greenland shark, as the sharks’ consciousness life cycle is more ‘linear’. Perhaps one organism starts out in one region of the model and develops into another with vastly different conscious experiences throughout its life cycle.

#### Velocity of change

We may consider the velocity at which the variations in sentience profiles occur. We might find a species-typical velocity usable, perhaps, for welfare assessments. For example, we might find a correlation between an organism having an outlier velocity and having low welfare, i.e. consciousness may change too slowly or too rapidly.

An example of the foregoing might be boredom in captive animals (I thank the reviewer for this suggestion.). It has been suggested that some animals may experience boredom, especially when confined to monotonous or impoverished environments (Burn 2017, Meagher 2019). The feeling of boredom may be caused by an abnormal velocity in the extent to which some dimensions are satisfied over time, in particular, Evaluative Richness. If so, it may be possible to compare the velocity of change along some dimensions for bored animals to the velocity of change of closely related states, such as frustration and apathy, in the hope of distinguishing between them more clearly.

#### Dependency relations

By comparing the variations in temporal profiles over time, it becomes possible to find dependency relations between the dimensions. We can determine whether some dimensions are closely connected, such that when one increases or decreases, the same or the opposite occurs for the other. We are also able to compare the dependency relations between dimensions of different species. Perhaps they have similar relations, which could lead to important insights into a commonality of dependencies among conscious entities, ‘or’ we may discover little to no similarity in dependency relations, which would indicate that the dimensions differ in their interconnectedness among conscious entities. This means that two new possibilities arise: (i) finding the dependency relation between the dimensions ‘within’ a species and tracking whether they change over time; (ii) comparing the dependency relations between the dimensions ‘across’ species. Do they have a similar pattern of change in dependency over time? For example, it may even be the case that every time the Unity dimension increases, independent of species, one or more dimensions also increase (or decrease), while the specific dimensions that increase (or decrease) differ among species, which would suggest that Unity has species-typical dependency relations.

For example, if we were to discover that low Unity correlates with low Temporality, this would provide us with some evidence of a common underlying structure on which both capacities depend. Furthermore, and perhaps most importantly, it may turn out that some dimensions dissociate in non-human organisms. Some dimensions may have a dependency relation in humans, which one might mistakenly take to be necessary for certain consciousness-related capacities. Yet, if the capacities are realized without the dependency relation in other entities, this would be evidence that those capacities may be expressed in various forms.

Let me summarize what I have argued so far. The advent of a multidimensional model of GSC is a recent advancement in consciousness science and philosophy of mind. In keeping with McKilliam (2020), I have argued for a capacities account of GSC and sentience. I have argued that by modelling the ‘ontogeny’ of consciousness, new types of comparisons become possible. I have attempted to illustrate this by modelling the changes in the sentience profile of a butterfly life cycle, showing the dramatic changes in sentience that may occur throughout the life cycles of conscious entities. This example allows us to see how we can compare the degrees of variation in consciousness, the various velocities at which the variations occur, and what the dependency relations of the capacities might be.

Those who advocate the multidimensional framework argue that a unidimensional framework leaves out important information about consciousness (Bayne et al. 2016, Birch et al. 2020). Given this, they too should abandon the idea that single profiles sum up the experiential expanse of an organism, as such representations also leave out vital information about consciousness. I have argued that there are benefits to moving away from speaking of ‘the’ consciousness of organisms as summed up in a single profile, to speaking of developments of consciousness.

I now turn to the second case, where I will discuss the multidimensional approach to GSC and focus specifically on DoC. As in the case of sentience profiles, I will construct temporal profiles to illustrate how tracking changes in the form of fluctuations in patients’ capacities, brings with it new modes of diagnosis and avenues for scientific and philosophical research, and emphasizes the dynamic approach already prevalent in the study of DoCs.

## Case 2: disorders of consciousness

In this section, I turn to DoC. I will introduce the multidimensional framework of DoCs by Bayne et al. (2017) but, like in the case of the sentience model, I will expand it by modelling the developments of changes in a patient’s condition. This leads me to discuss different types of fluctuations that DoC patients may produce which may help with both diagnosis and prognosis.

### Kinds of DoCs and diagnosis

DoCs are states of consciousness that can result from brain damage. These states include coma, UWS, sometimes called the VS, minimally conscious state (MCS), which has been divided into MCS^+^ and MCS^-^, and emerged from minimally conscious state (EMCS). Such states are characterized by a severe impairment of normal behavioural and communicative capacities.

DoCs have been described as having much in common with altered states of consciousness such as REM-dreaming, delirium, extreme drowsiness, and anaesthesia (Bayne and Hohwy 2014, p. 130), as those states, too, are states of disruption to systems responsible for voluntary behaviour. Some have even suggested that schizophrenia (Giersch and Mishara 2017) and major depressive disorder (Whiteley 2021) should be considered DoC.

Perhaps the most familiar DoC is coma. To be in a coma is to be in a state of unarousable unawareness, which usually lasts about 2 weeks (Peterson 2016, p. 2). In this state, the patient has no sleep/wake cycle and may be unable to breathe on their own, and need to be kept alive by artificial hydration, nutrition, and ventilation. UWS is characterized by a sleep/wake cycle, but the patient is still unresponsive to auditory and tactile stimuli (Laureys 2005, Peterson 2016, p. 2).

In the minimally conscious state (MCS) there is a sleep/wake cycle and some behavioural responses are present. Recently, this state has been divided into two distinct states: MCS^-^ and MCS^+^. MCS^-^ describes low-level behavioural responses such as visual pursuit, localization of noxious stimulation or contingent behaviour, such as appropriate smiling or crying in response to emotional stimuli (Bruno et al. 2011, p. 1375). MCS^+^ describes high-level behavioural responses, such as command following, intelligible vocalization, and non-vocal communication (Bruno et al. 2011, p. 1375). Lastly, in emergence from the minimally conscious state (EMCS) functional interactive communication, functional use of various objects, writing, and yes/no signals are observed.

Importantly, at each of the above-mentioned stages, the individual may transition to another stage or transition directly to full consciousness. A coma patient does not necessarily progress through all the DoCs to become fully conscious. Furthermore, it is possible for a patient in UWS to transition to MCS, and from MCS to lapse back into UWS (Giacino et al. 2009, p. 37).

**Figure 5:**
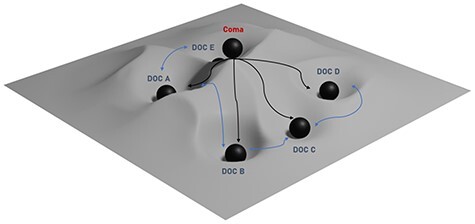
A hypothetical DoC-state space, where the gradient between two points represents the transition probability between the two states. Coma may be an unstable state, owing to patients’ relatively quick transition from that state. Types of DoCs not identified here by specific names are the valleys. Arrows stemming from the coma state illustrate the possible paths a patient may take when coming out of coma. Arrows between DoCs indicate the possibility of patients’ transitions between DoCs.

It might be useful to think of DoCs as states in a state space where valleys are attractor states associated with kinds of DoCs and peaks are unstable states (Fig. 5). A person who suffers a traumatic brain injury that puts them into a coma will be transported to a peak within the state space. From this peak the patient may progress to any number of DoCs, or back to full consciousness or brain death.

There is a growing body of literature surrounding the difficulty of distinguishing the various DoCs (Bayne et al. 2017). This is especially apparent for UWS and MCS (Giacino et al. 2002, Sergent et al. 2017, Kondziella et al. 2020) which is borne out by the high rate of misdiagnosis of MCS patients as UWS, the common estimate of misdiagnosis being approximately 40% (Young et al. 2021, p. 3297).

Bayne et al. (2017) argue that there are at least two major problems with our current taxonomy of DoCs, making it inadequate for medical science. The two problems are that (i) the categories of DoC do not take aetiology into account; (ii) the current taxonomy relies mostly on capturing differences in consciousness on purely behavioural grounds, exemplified in the Coma Recovery Scale-Revised (CRS-R), which is the standardized assessment tool for diagnosing a patient’s DoC. This scale, and others like it, use behavioural assessments of the patient, and test their auditory, visual, and motor skills, among other things.

Starting with (i), Bayne et al. state that the cause of a patient’s DoC plays an important role in the prognosis, which in turn influences decisions regarding life termination. For example, patients who are UWS because of a traumatic brain injury have a greater chance of transitioning to MCS than patients in UWS that are not the result of a traumatic brain injury. Therefore, we should include aetiology in our assessments of patients’ DoC.

Regarding (ii), technology such as EEG and fMRI provide new methods for assessing patients, which reveal that some patients are covertly conscious, i.e. they are conscious but produce no behavioural indicators of this at bedside assessments, such as those made when using the CRS-R. In a series of seminal studies, UWS patients and healthy volunteers were asked to imagine playing tennis while in an fMRI. The researchers discovered that the UWS patients could command-follow, and that their neural responses were indistinguishable from the healthy volunteers (Owen et al. 2006, Boly et al. 2007):

In sum, there is now overwhelming evidence that significant numbers of VS patients—that is, patients who are *correctly* diagnosed as VS according to current clinical guidelines—are conscious. Indeed, in some cases, their conscious capacities rival not just those of MCS, but also those of EMCS patients. Even more profound disruption to the current DoC taxonomy is suggested by evidence of covert consciousness in some comatose patients. (Bayne et al. 2017, p. 868).

It is issues such as these that have led some to argue that we should rethink our DoC taxonomy (Bayne et al. 2017, Kondziella et al. 2021). Bayne et al. (2017) suggest that the current taxonomy needs to be radically revised, proposing a way forward with the introduction of a multidimensional framework, like the one for GSC by Bayne et al. (2016), but specifically for DoCs. Their approach reforms the current taxonomy of DoCs by replacing the current, discrete categories with ‘categories that are graded and represent regions in a multidimensional space’ (Bayne et al. 2017, p. 870). They state that this approach would combine both behavioural and brain-based criteria (such as fMRI) when categorizing DoCs, and a multidimensional model framework that captures patient performance of both cognitive and behavioural tasks. I regard this approach as a step in the right direction. Just as in the case of the temporal sentience profile, I will construct an illustrative example of a temporal DoC profile and show that doing so may offer benefits for both diagnosis and prognosis.

### DoC as dynamic kinds

DoC patients fluctuate. Not only do some patients have a sleep/wake cycle, but they may also become hungry, may be in pain, in distress, or be tired. Medication and the environment may influence levels of arousal, and spontaneous brain activity may occur. These are just some of the fluctuations that can take place in a patient, and are some of the reasons patients are misdiagnosed (Peterson 2016, Young et al. 2021) which has led to recommendations for repeated assessment (Wannez et al. 2017, Kondziella et al. 2020, Wang et al. 2020, Da Conceição Teixeira et al. 2021):

[A] patient’s diagnosis might be inaccurate due to fluctuation or evolution in her condition. Cognitive fluctuations are common in patients with DoC, yet a clinical exam only represents a snapshot of the patient’s condition in time. Serial assessment of a patient addresses this issue, but requires time and resources that are often unavailable, especially in acute settings. (Young and Peterson 2022, p. 382).

In one study, Wang et al. (2020) conducted repeated assessments of a sample of 89 patients diagnosed as UWS (VS). On re-examination, this number was reduced to 66, and on further examination, that number was further reduced to 54:

(…) the difference between the results of single assessments and repeated assessments emphasizes that the fluctuations of patients’ responsiveness have an effect on neuro-behavioral assessments, and also emphasizes the importance of repeated assessments in clinical diagnosis. (Wang et al. 2020, pp. 5–7).

These fluctuations are noise in the signal that complicates proper diagnosis, but is that so for all fluctuations? I argue that some fluctuations may be treated as kind-identifiers, rather than pure noise. If so, then some of the fluctuations of DoC patients should be accounted for by Bayne et al.’s (2017) multidimensional model of DoCs. To better grasp the ways in which patients fluctuate, I will distinguish between three types of fluctuations: inter-DoC fluctuations, intra-DoC fluctuations, and DoC-defining fluctuations. Inter-DoC fluctuations are fluctuations ‘across’ regions associated with kinds of DoCs in the multidimensional model. Intra-DoC fluctuations are fluctuations ‘within’ regions of the model. And, as the name suggests, DoC-defining fluctuations may be used to define kinds of DoC. The types of fluctuations mentioned above, such as changes in hunger and distress, are intra-DoC fluctuations.

#### Inter-DoC fluctuations

This type of fluctuation is found in patients whose recovery from coma to consciousness takes them through one or more DoCs. As such, inter-DoC fluctuations are fluctuations ‘between’ kinds of DoC. An example of this is found in Giacino and Trott (2004), whose patient transitioned from coma to UWS, to MCS, lapsed back into UWS, and then recovered to MCS: ‘Our patient recovered signs of consciousness at approximately 2 months postinjury, unexpectedly lapsed back into VS after 3 months, and eventually regained consciousness again in month 4’ (Giacino and Trott 2004, p. 263). Furthermore, Majerus et al. (2005) note that the severe changes in DoC patients is not uncommon, especially during recovery:

Assessment of awareness is not a matter of all or nothing. Recovery of awareness is a very gradual process, with sometimes great leaps forwards, but more often subtle changes, and also sometimes setbacks. For the patient emerging from coma, it is of utmost importance that the medical staff adapts its assessment to the level of awareness the patient is currently in. The subtlest signs of awareness, as well as their fluctuation, have to be reliably captured as they are the only means for avoiding misdiagnosis, but also for communicating with these patients. (Majerus et al. 2005, p. 411).

This type of fluctuation is illustrated by the blue arrows in Fig. 5. It is unclear whether there is a pattern to this type of fluctuation, i.e. whether patients, once in one state, are more likely to move from that state to another state.

#### Intra-DoC fluctuations

The second type of fluctuation occurs ‘within’ a kind of DoC, rather than between them, i.e. within the region of the multidimensional model related to a kind of DoC.

Intra-DoC fluctuations occur in both UWS and MCS patients. When they tested the auditory and visual performance of chronic UWS and MCS patients using the Coma Recovery Scale-Revised (CRS-R), Cortese et al. (2015) demonstrated that the patients’ test scores varied according to when during the day the tests were administered. Both UWS and MCS patients had higher scores in both categories in the morning than in the afternoon. The study concluded that UWS patients had as much as a 30% greater risk of being misdiagnosed as MCS in the morning than in the afternoon, at least once during the weeks in which the study took place, precisely because of the fluctuations in their auditory and visual scores:

In this respect, the CRS-r reliability (…) is not to be questioned on the ground of its variability during the day, nor is to be questioned the examiners’ accuracy. Instead, the observed CRS-r differences between morning and afternoon are likely to reflect individual changes in the subject’s level of visual, auditory and motor functioning conceivably due to changes in the neuronal/non-neuronal factors that modulate the brain state. (Cortese et al. 2015, p. 5).

Importantly, Intra-DoC fluctuations are treated as noise in the signal. The assumption is that the patient is in a specific DoC and, due to fluctuations in arousal, they are at risk of being misdiagnosed. However, there is a separate way in which fluctuations are understood in the literature, namely as a defining feature of kinds of DoC, specifically for MCS.

#### DoC-defining fluctuations

**Figure 6:**
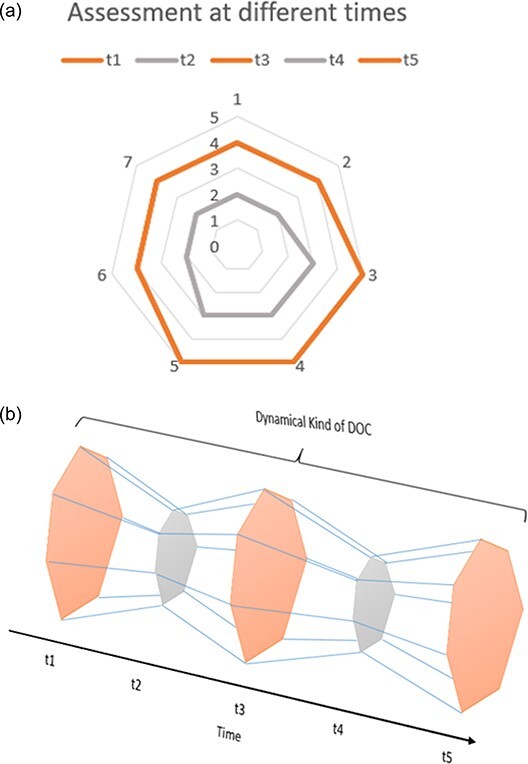
(a) A patient is assessed at several points in time. (b) A pattern of fluctuation becomes apparent, indicated here by the different profiles.

The third type of fluctuation is used as a defining feature of a DoC. When Giacino et al. (2002) introduced the MCS to the literature on DoCs, they defined MCS in fluctuating terms: ‘In MCS, cognitively mediated behaviour occurs inconsistently, but is reproducible or sustained long enough to be differentiated from reflexive behaviour’ (351). Thus, part of what identified patients as being in MCS was that they ‘inconsistently’ displayed cognitively mediated behaviour, i.e. the kind—MCS—is not stable, but fluctuates. This is important because the claim being made is that at least one DoC is defined in relation to change understood as being defined partly by its temporal progression. We cannot capture the kind at a moment in time because the kind is not wholly present at any moment. Here we see that fluctuations in the kind identified by Giacino et al. (2002) were understood not as noise, but as information. This shows that, at least for MCS, a single profile in the multidimensional space does not properly capture the changes in the consciousness-related capacities. To illustrate this, assume we assess a patient several times (Fig. 6a), yielding a temporal DoC profile (Fig. 6b).

In Fig. 6, the DoC kind ‘is’ the fluctuating pattern of orange to grey to orange to grey ‘with all that lies between’. This means that not only might DoCs be multiply realizable, so that we start to associate a region within the multidimensional model as expressing a kind; at least some DoCs are also extended over time.

What the temporal DoC profile makes clear is that the profiles have vague boundaries. Just as in hominin evolution, where no clear line separates *Homo habilis* from *Homo erectus*, there is no clear instance where one profile becomes another. However, the transitions between the various profiles in the temporal profile need not be as smooth as depicted. One process that may aid in differentiation is phase transitions. There may be rather radical changes from one profile to another over a short time span. Such instances of criticality would function as clearer boundaries between DoCs, moving the subject from one attractor state to another.

Importantly, intra-DoC fluctuations occur simultaneously with DoC-defining fluctuations. MCS patients are defined as such by their specific form of inconsistent ability to produce certain cognitively mediated behaviour (Giacino et al. 2002), a type of fluctuation I have called DoC-defining fluctuations. MCS patients also show intra-DoC fluctuations in their visual and auditory capabilities (Cortese et al. 2015). This means that there is an overarching pattern of fluctuation that is taken to be information about the patients’ DoC, and another type of fluctuation that is taken to be noise, which increases the risk of misdiagnosis. Furthermore, and just as important, in time we may start to see some Intra-DoC fluctuations as DoC-defining fluctuations, meaning that some fluctuations that we currently treat as noise using CRS-R may turn out to be indicative of kinds of DoC. For example, it may be that there is an underlying pattern of change in the changes in auditory and visual performance in chronic MCS and UWS patients, which indicates their DoC. Or, being tired, although it produces fluctuations in capacities, may not be indicative of a specific kind of DoC, but a fluctuation in tiredness over time might.

Examples of the foregoing are already beginning to emerge, as in recent studies researchers have attempted to use EEG to identify new patterns of change in DoC patients, as they attempted to correlate those patterns with a patient’s CRS-R diagnosis (Piarulli et al. 2016, Naro et al. 2018, Bai et al. 2021). Put differently, in time we may learn to identify the information (DoC-defining fluctuations) in the noise (Intra-DoC fluctuations).

With this emphasis on fluctuations, we can see that the terminology that McKilliam (2020) introduced in his capacities account of GSC needs to be expanded to include developmental changes. For example, if a healthy person has a severe accident and suffers a DoC, they have lost some of their capacities. It would be a mistake to say that their capacities are simply ‘offline’. The capacities would have to be redeveloped so that they could switch between being online and offline.

Lastly, one might worry that there is a friction between temporally extended kinds of DoC and fluctuations between kinds of DoC. For example, what if only a section of a dynamic kind is expressed before a patient transition into another kind? How much of the fluctuating pattern must be present before we take a kind to be present?

Let us take the case of a patient whose condition evolves, progressing to new states and lapsing into former ones. If we understand temporally extended kinds like they were symphonies, the patient’s progression may be understood as ‘sounding like’ or ‘playing part of’ various symphonies. Symphonies have definite structures, consisting of a series of movements. Although listening to one movement is not the same as listening to the whole symphony, it does involve listening to a ‘part’ of the symphony. What I am suggesting here is that the various transitions of recovering patients may consist of something similar to an amalgamation of movements from various symphonies. First, the patient may express the second movement of Symphony A, then the fourth movement of Symphony B, and so on. They would not be expressing the dynamic kinds, but subsets of those kinds. They would be ‘sounding like’ different kinds.

### New modes of comparison

As with the temporal sentience profile, the temporal DoC profile allows us to compare modes of variation, velocity of change, and dependency relations among the various dimensions. The temporal DoC profile illustrates the possible importance of the ‘ways’ in which a stably fluctuating patient fluctuates. There may be typical and atypical changes in one’s DoC, and these changes may occur at typical or atypical rates. By focusing on temporal variation, we can start to measure the velocity of fluctuations. Tracking another patient deemed to be in a similar state of DoC may reveal that, once their fluctuations are compared, one patient has a greater degree of variability than the other, i.e. there may be significant differences in the extent of change or their rate of variation may differ.

This focus on fluctuations should not be only for patients with DoC-defining fluctuations, but also those undergoing inter-DoC and intra-DoC fluctuations. For patients who rapidly and unreliably transition between DoCs, tracking the degree and velocity of change may provide valuable information for prognosis. There may be a difference in the rate of transition between patients who transition from coma to a chronic DoC and those who transition from coma to a DoC, which they inhabit briefly, i.e. just as aetiology plays a role in prognosis, so too may the ‘way’ a transition occurs give us new insights useful for prognosis.

To summarize, inter-DoC fluctuations are fluctuations or evolutions across kinds of DoC, intra-DoC fluctuations are fluctuations within kinds of DoC, and DoC-defining fluctuations are defining features of kinds of DoC. DoC-defining fluctuations, unlike Intra-DoC fluctuations, take fluctuations not as noise in the signal, but as the signal itself. If we start to shift more deliberately to a view of fluctuations as possibly providing valuable information, instead of treating them mainly as hurdles to be overcome, new modes of comparison will present themselves with the potential to aid not only diagnosis, but also treatment.

Patients who suffer from DoC should be evaluated within a multidimensional framework that considers aetiology and uses both behavioural tests and brain scans to test for both overt and covert signs of consciousness. Also, I argue that the patients’ inter-, intra-, or the DoC-defining fluctuations should be analysed and used, as they too may reveal hidden patterns that may be useful not only for diagnosis, but also for prognosis and treatment.

## Conclusion

I have argued that modelling changes in DoC and organism life cycles introduces new modes of comparison among organisms and among kinds of DoCs. Modelling the consciousness ‘life history’ of organisms and the fluctuating patterns of DoC patients allows us to investigate degrees of variation, the velocity with which such variation occurs, and to gain insight into the interconnected web of dependency relations between the many consciousness-related capacities.

Three points have been made in this paper. The first point is that both GSC and sentience profiles change over time, and therefore, we need to expand McKilliam’s (2020) capacities account to include the developments of conscious-related capacities. The capacities account as it was originally formulated may work for seemingly stable systems, such as healthy adult humans, but because it cannot properly account for the development, loss, redevelopment through recovery, and fluctuations of capacities, we should expand this framework. The second point is that modelling the ‘life history’ of consciousness of organisms is pragmatically useful, as new modes of comparisons between species emerge. The third point is that modelling changes in DoCs within a multidimensional framework also allows for new modes of comparison and shifts our focus to the various ways in which DoC patients’ conditions may fluctuate.

We should refrain from thinking that we can ‘capture’ the consciousness of any organism with a single profile. Organisms have life cycles, and therefore have changing profiles of consciousness. I have argued that this is also the case for at least some DoCs. By not modelling developments, tracking changes in consciousness over time, we risk leaving out too much information about consciousness. We should emphasize the dynamic aspect of consciousness, not set it aside, in an effort to find new ways of studying consciousness, both across species and within individuals.

## Data Availability

No new data were generated or analysed in support of this research.
